# Israel’s response to the COVID-19 pandemic: tailoring measures for vulnerable cultural minority populations

**DOI:** 10.1186/s12939-020-01191-7

**Published:** 2020-05-19

**Authors:** Ruth Waitzberg, Nadav Davidovitch, Gideon Leibner, Nadav Penn, Shuli Brammli-Greenberg

**Affiliations:** 1 The Smokler Center for Health Policy Research, Myers-JDC-Brookdale Institute, JDC Hill, P.O.B. 3886, 91037 Jerusalem, Israel; 2 Department of Health Systems Management, School of Public Health, Faculty of Health Sciences, Ben-Gurion University of the Negev, Beer-Sheva, Israel; 3 Department of Health Care Management, Faculty of Economics & Management, Technical University Berlin, Berlin, Germany; 4 The Hebrew University, Hadassah Medical School, Jerusalem, Israel; 5 Department of Health Administration and Economics, Braun School of public health, Faculty of Medicine, the Hebrew University of Jerusalem, Jerusalem, Israel

**Keywords:** COVID-19, Health policy, Cultural minorities, Ultra-orthodox Jews, Ramadan

## Abstract

Every country has vulnerable populations that require special attention from policymakers in their response to a pandemic. This is because those populations may have specific characteristics, culture and behaviours that can accelerate the spread of the virus, and they usually have less access to healthcare, particularly in times of crisis. In order to carry out a comprehensive national intervention plan, policy makers should be sensitive to the needs and lifestyles of these groups, while taking into account structural and cultural gaps.

In the context of Israel, the two most prominent and well-defined minority groups are the ultra-Orthodox Jewish community and parts of the Arab population. The government was slow to recognize the unique position of these two groups, public pressure eventually led to a response that was tailored to the ultra-Orthodox community and during the month of Ramadan a similar response has been implemented among the Arab community.

## Background

The response to the COVID-19 pandemic in high-income countries and the public discourse surrounding it have focused on the majority population group but they have been inadequate in addressing the special conditions and needs of minority groups. Minorities have been shown to experience greater challenges in adopting physical distancing measures and they face barriers in gaining access to information, testing and healthcare services relative to the majority population, particularly during emergencies. It is likely that minorities face additional risk from COVID-19 based on their unique characteristics, culture, and position in society, together with less than optimal health status and the discrimination they face. This may generate additional health risk during this pandemic, not just for minorities themselves but for society as a whole [1]. About one-third of Israel’s population is composed of various types of minority groups and therefore can serve to draw lessons for addressing minority issues during COVID-19.

Israel entered the pandemic with a robust economy. It has a National Health Insurance (NHI) law that grants universal coverage of healthcare services to all residents including primary and secondary care in the community, hospital services, pharmaceuticals and diagnostic exams [2]. The Israeli healthcare system has an effective community and primary health care system; however, its hospital system is overburdened and crowded, with lower rates of hospital beds and nurses compared to the OECD average (2.2 curative beds and 5 practicing nurses per 1000 population compared to 3.6 and 8.8, respectively in 2017) [3]. Medical wards, in which COVID-19 patients are treated, suffer from a more severe shortage of beds and medical professionals than surgical wards. About 619 of the hospital beds (4.2%) are located in ICUs [4]. This is in addition to persistent inequalities in access to care between higher and lower socio-economic status (SES) individuals, and between those living in the center of the country and those in remote areas in the north and south of the country despite the NHI’s universal coverage [5, 6].

Israel responded rapidly and on a national level during the prevention phase of the pandemic: it closed its borders relatively quickly after the outbreak in China and quarantined travellers returning from high-risk areas. The containment phase included the quarantine of diagnosed individuals, but the capacity for testing suspected cases (in order to quarantine them) was slow to ramp up. The mitigation phase was also implemented with dynamic measures, with policy being adapted on almost a daily basis. This included physical distancing, which eventually was extended to a lockdown. Leading the response is the Ministry of Health (MoH), which coordinates surveillance, communication and reporting efforts. In addition, a National Corona Information and Knowledge Centre was established, which provides up-to-date information on the development of the pandemic (in Hebrew).

Nonetheless, vulnerable cultural minority groups became a focus of COVID-19 infection due to their unique socioeconomic and cultural characteristics and their previously existing low levels of access to healthcare services. This required tailored measures first to respond to their needs and then also to mitigate the spread of the virus.

## Israel’s response to the COVID-19 pandemic

The MoH issued official directives on how to reduce the risks of COVID-19 infection on February (https://govextra.gov.il/ministry-of-health/corona/corona-virus-en/). These were disseminated in 10 languages, both through the conventional media and on social media platforms.

Starting from February 28th, the MoH published epidemiological reports that included flight details and places that each infected person had frequented up until diagnosis and called on all individuals who had been in contact with a diagnosed person to self-quarantine for 14 days.

As the number of cases increased, the MoH started using big data and technological means to improve and speed up investigations (starting on March 25th). Mobile phone applications and websites are now in use to collect spatial data on the whereabouts of diagnosed individuals, and anyone who had been in contact with them were instructed to self-quarantine. While public discussion has begun on privacy issues and the proportionality of the means being used, and has even reached the Supreme Court, sometimes these means are not particularly relevant in the case of vulnerable population groups, as they do not use or have less access to these applications, internet and websites.

The government declared a lockdown starting on March 25th (when there were about 2500 cases). The lockdown added several restrictions and legal sanctions to the previous guidelines. People were urged to stay at home; many workers were temporarily laid off and others began working from home; individuals were prohibited from going farther than 100 m from their homes; and gatherings of over 10 people were prohibited. Violation of these regulations is a finable offence. Starting from April 12th, it became mandatory to wear a mask in public.

## Responses tailored to vulnerable cultural-minority populations

Policymakers should focus on vulnerable minority groups in view of their unique characteristics, needs, culture and behaviours, which can hinder the implementation of physical distancing (such as, for example, the density of the communities they live in), and therefore accelerate the spread of the virus to a faster rate than among the mainstream population. Furthermore, since minorities usually have low SES, live in remote areas, and have language barriers, they have less access to healthcare services, including testing, particularly in times of crisis, even in countries with universal coverage due to lack of ability to pay and lower availability of services as well as cultural differences. If countries fail to take this into account in their policymaking, vulnerable minority groups will provide particularly fertile ground for the spread of COVID-19 to other sectors and they will be blamed for exacerbating the crisis. Therefore, it is imperative to formulate a comprehensive national intervention plan that is sensitive to the needs and lifestyle of these groups while at the same time fostering their trust in the government and its institutions.

In Israel, the ultra-Orthodox Jewish community and the Arab population are the most prominent and well-defined minority groups, representing 12% [7] and 20% [8] of the total population, respectively. During March 2020, cities predominantly inhabited by ultra-Orthodox Jews became foci of the COVID-19 outbreak. After some delay, the government implemented special measures adapted to this population. By mid-April, higher rates of COVID-19 infection were also reported in Arab cities, and there is a concern that these will also become the source for an acceleration of the outbreak and will require specifically tailored measures.

Ultra-Orthodox Jews are characterized by large families (in 2017, their fertility rate was 7.1 children per woman), a low SES, crowded living conditions, an intense social and community life, and higher dependency on public transportation – factors that provide an ideal environment for COVID-19. Furthermore, this population is characterized by low rates of formal labor force participation [9] and therefore are not always entitled to the economic assistance from the state related to the pandemic, such as unemployment benefits. Despite Israel’s universal National Health Insurance, the ultra-Orthodox community’s access to healthcare was lower than that of the general population even before the pandemic, due to the distances from healthcare providers, language and cultural barriers, and sometimes also the inability to pay copayments [10], and this situation did not improve during the pandemic.

Apart from socioeconomic characteristics, the ultra-Orthodox also have different behavioural norms that limit their ability to comply with mitigation measures. For example, some groups within the ultra-orthodox community do not use the conventional media nor social media, which the MoH has used to disseminate information and directives. Therefore, they are less informed about measures such as physical distancing while the information they did receive was not tailored to the community in terms of language and content. In addition, many members of this community, and in particular women, do not use smartphones and did not receive SMS messages alerting them of contact with an infected individual or they suffered from language barriers in order to understand the messages. Thus, this community did not self-quarantine to the extent that other populations did. Finally, ultra-Orthodox Jews live in tight-knit communities that tend to follow the instructions of their own leadership over complying with government directives.

As a result, the rate of the COVID-19 morbidity in cities primarily inhabited by ultra-Orthodox Jews is higher than that of the general population [11]. Thus, the government came to the realization that measures were needed in these locations that are tailored to this community.

The first measure taken by the government was to build trust among the leadership of the ultra-Orthodox community and to gain their cooperation in, for example, communicating the importance of good hygiene and physical distancing. In addition, existing civic society networks and structures, such as aid and charity institutions, were recruited in order to provide essential services and goods such as food and prescription drugs during the lockdown, in cooperation with the health and welfare services and local municipalities.

Nonetheless, a great deal of effort was expended and precious time lost until the MoH, police and local authorities were able to create effective communication channels and to convince the ultra-Orthodox leadership to mandate physical distancing in their communities. For example, the education system in Israel was shut down on March 12, but some ultra-orthodox educational institutions continued operating for a full week after that. Although physical distancing measures were put in place in early March, synagogues were instructed to shut down only 2 weeks later, on April 19. Even then, a few ultra-Orthodox religious leaders permitted their followers to continue praying in synagogues for another 10 days (until April 31), even after it became evident that ultra-Orthodox cities and neighbourhoods were becoming foci of the virus’ spread. It is also worth mentioning that once the leadership instructed the community to follow the directives, the community complied almost without exception.

The second measure implemented by the government in ultra-Orthodox areas was increased diagnostic testing, isolation of positive individuals, and the evacuation of the elderly to now-empty hotels rented by the government.

The third measure was the quarantine of cities and towns with high infection rates. Thus, on April 1st, the government approved special emergency regulations to implement stricter physical distancing measures in these cities, the majority of which were ultra-Orthodox, and to quarantine them from the rest of the country. On April 12th, the measures were extended to neighbourhoods in the large cities with high infection rates. For example, Jerusalem was divided into four quarters and residents were restricted to their quarter. The army was called in to collaborate with the police in evacuating the elderly and families that agreed to do so, enforcing the quarantine of cities and neighborhoods, and distributing food and essential provisions to residents, particularly those in disadvantaged neighborhoods.

### Measures during the Passover holiday

Holidays are critical occasions characterized by family get-togethers, increased travel, and greater attendance of religious services, all of which can potentially accelerate the spread of COVID-19. In order to curtail these activities, the government declared special measures during the Passover period (April 8 to April 15), including tighter restrictions on mobility and public gatherings. Thus, movement between Jewish cities was forbidden for 3 days starting from April 7. On April 8th and 9th people were prohibited from leaving home, even to buy food. In order to enforce these measures, the police set up roadblocks on major routes between cities. At the same time, the government approved a special one-time children allowance for Passover in the amount of 500 NIS (~USD125) per child and up to 2000 NIS per family, without any means tests and a 500 NIS grant for the elderly (for which the criteria have yet to be publicized).

## Discussion and conclusion for policymakers

Responding to the COVID-19 pandemic requires a concerted effort from the entire population. There are certain groups in every country that are less accessible due to language, culture and access barriers and as a result they do not fully participate in the prevention and containment efforts. In Israel, these are primarily the ultra-Orthodox Jews. Cities and neighborhoods with high concentrations of ultra-Orthodox Jews became foci of the outbreak and required specially tailored mitigation and containment measures. This involved more stringent social distancing measures, alongside close coordination with religious leaders.

Similarly, responses to the COVID-19 pandemic in the Muslim population have been adapted to that population in order to avoid an emerging focus of infection. As in the case of the ultra-Orthodox community, the Arab population is not a monolithic group with respect to urban vs. rural, socioeconomic status, etc., and furthermore many in this community work in the healthcare system and thus are at high risk. The Bedouin, many of whom live in unrecognized settlements in southern Israel (the Negev) are particularly vulnerable. Some parts of Arab society share some characteristics with ultra-Orthodox Jews, including low SES and an intense social and community life. However, in contrast to the ultra-Orthodox, many Arabs live in rural and remote areas and they typically comply with public health norms (for example, vaccination rates are high in this community), two traits that support containment and mitigation measures. Policy-makers have taken into consideration the month of Ramadan, which takes place in 2020 from April 23 to May 23, a period in which traditional Muslims gather to pray and feast. After the experience with the Ultra-Orthodox Jews, the MoH and the Arab community’s religious leaders have been planning specific measures to maintain physical distancing during this period. These should be accompanied by improved access to healthcare, increased diagnostic screening, stringent quarantining of infected individuals, and humanitarian responses, such as the provision of food and holiday-related items.

There is a particular challenge in providing information, screening and healthcare to dispersed Bedouin communities in remote areas who do not have good access to healthcare.

Israel’s experience in dealing with its special populations can serve as an example to other countries in how to adapt public health measures to fit the situations of marginal groups, culturally distinct populations, minority groups, etc. These populations require specially targeted and tailored responses that take into account their situation with regard to access to healthcare, living and working conditions, and ability to maintain physical distancing, so as not to become foci of infection that will ultimately affect all sectors of society.

Apart from taking into account the needs and lifestyles of these groups, it is important to foster their trust in the government and its institutions and to work closely with their leadership in order to ensure compliance with COVID-19 mitigation measures. No less important is to provide sufficient economic safety nets for poor families in these communities. Public health measures must carefully take into account the effects of physical distancing and lockdowns in poor communities, such as worsening the already-existing education gaps, food insecurity and levels of violence [1, 12].

The mortality rate from COVID-19 is relatively low in Israel (26.45 deaths per 1 million population as per May 5th, 2020 [13]), which can be attributed to a number of factors: the rapid and comprehensive response of the Israeli government, the young age distribution of the population, the high level of compliance with physical distancing among most the general population, and the tailored measures implemented among minority populations. The effect of the government’s measures on patients suffering from other conditions who had to delay treatment and the increased financial burden on businesses will become clearer in the coming months and years. Here as well there is a challenge to take vulnerable groups into account in the formulation and implementation of public health measures against COVID-19.

## Data Availability

Data sharing is not applicable to this article as no datasets were generated or analysed during the current study.

## Abbreviations

COVID-19: Corona Virus December 2019
NIS: New Israeli Shekels
USD: US Dollars
NHI: National Health Insurance
SES: Socio-economic status
